# Exosomal circ_0008285 in follicle fluid regulates the lipid metabolism through the miR-4644/ LDLR axis in polycystic ovary syndrome

**DOI:** 10.1186/s13048-023-01199-x

**Published:** 2023-06-15

**Authors:** Li Yu, Chen Wang, Doudou Zhang, Miao Liu, Te Liu, Baishen Pan, Qi Che, Suying Liu, Beili Wang, Xi Dong, Wei Guo

**Affiliations:** 1 Department of Laboratory Medicine, Zhongshan Hospital, Fudan University, No. 111 Yi Xue Yuan Road, 200032 Shanghai, PR China; 2 Reproductive Medicine Center, Zhongshan Hospital, Fudan University, No. 250 Xiao Mu Qiao Road, 200032 Shanghai, PR China; 3 Shanghai Geriatric Institute of Chinese Medicine, Shanghai University of Traditional Chinese Medicine, No.725 South Wan Ping Road, 200031 Shanghai, PR China; 4 Department of Laboratory Medicine, Xiamen Branch, Zhongshan Hospital, Fudan University, No. 668 Jin Hu Road, 361015 Xiamen, PR China; 5 Department of Laboratory Medicine, Wusong Branch, Zhongshan Hospital, Fudan University, No.216 Mudanjiang Road, 200940 Shanghai, PR China; 6 Cancer Center, Shanghai Zhongshan Hospital, Fudan University, No. 111 Yi Xue Yuan Road, 200032 Shanghai, PR China; 7 Branch of National Clinical Research Center for Laboratory Medicine, No. 111 Yi Xue Yuan Road, 200032 Shanghai, PR China

**Keywords:** Polycystic ovary syndrome, Circ_0008285, MiR-4644, LDLR, Cholesterol metabolism

## Abstract

**Purpose:**

Exosomal circRNA, as an essential mediator of the follicular microenvironment, has been implicated in the etiological and pathobiological studies of polycystic ovarian syndrome (PCOS). This study aimed to determine abnormal circular RNA (circRNA) expression profiles in follicle fluid (FF) exosomes in patients with PCOS and identify the role of circ_0008285/microRNA (miR)-4644/low-density lipoprotein receptor (LDLR) axis in PCOS.

**Methods:**

Sixty-seven women undergoing IVF/ICSI, 31 PCOS patients and 36 non-PCOS patients were included in the cohort study. The circRNA expression profiles of FF exosomes in PCOS (n = 3) and control group (n = 3) were compared by RNA sequencing. In an additional cohort (PCOS:28 vs Control:33), the mRNA expression levels of four circRNAs from FF exosomes were further verified by qRT-PCR. Bioinformatic analysis and dual luciferase reporter gene assay verified the relationship between circ_0008285 and miR-4644 and between miR-4644 and LDLR. KGN cells were infected with sh-circ0008285 and transfected with miR-4644 mimic to verify their roles in lipid metabolism.

**Results:**

Four circRNAs showed significantly different expressions. Circ_0044234 was overexpressed in PCOS patients, while circ_0006877, circ_0013167 and circ0008285 were decreased in PCOS. Among four differentially expressed circRNAs, circ0008285 was enriched in lipoprotein particle receptor activity and cholesterol metabolism pathway by GO and KEGG pathway analyses. Luciferase assay confirmed the competing endogenous RNA (ceRNA) network circ_0008285/miR-4644 /LDLR. The intercellular experiments on circ_0008285 and its reduction in KGN cells showed that the consumption of circ_0008285 in exosomes could increase the expression of miR-4644 in recipient cells and inhibit the expression of LDLR, as well as increase free fatty acid secretion.

**Conclusion:**

Circ_0008285 can combine with miR-4644 to promote the expression of LDLR and affect the cholesterol metabolism of ovarian granulosa cells in PCOS. Our findings revealed the ceRNA network of circ_0008285 and provided a new path to investigate lipid metabolism abnormalities in PCOS.

**Supplementary Information:**

The online version contains supplementary material available at 10.1186/s13048-023-01199-x.

## Introduction

Polycystic ovarian syndrome (PCOS) is the most prevalent and complicated among endocrinopathies. PCOS is the cause of infertility in 5% to 10% of reproductive-age women [1]. It is a multi-factorial, heterogeneous syndrome with a variety of symptoms, including obesity, amenorrhea, hyperandrogenism, and menstrual irregularities [2]. An essential milieu for follicular growth and oocyte maturation is provided by follicular fluid (FF). Oocytes and the cells around them can communicate in both directions [3]. Exosomes are tiny, membrane-enclosed vesicles with diameters between 30 and 200 nm released by various live cells in both physiologically healthy and pathological conditions. Recently, a growing number of studies have demonstrated that exosomes are present in FF and function as message transmitters in intercellular communication by transmitting a range of proteins, lipids, miRNAs, and circRNAs [4–6].

The bulk of circular RNAs (circRNAs), which are covalently closed continuous loops that are stable across species, are conserved [7]. CircRNAs have been demonstrated to have a variety of biological roles, including miRNA sponges in controlling gene expression, as a novel class of abundant and persistent endogenous noncoding RNAs [8]. Numerous circRNAs were upregulated or downregulated in PCOS-affected individuals, according to findings from RNA sequencing (RNA-seq) [9–11]. In contrast there haven't been many studies focusing on exosome-mediated intercellular communication within the ovarian follicle. The roles of exosomal circRNAs in the development of PCOS are not well understood. Using RNA sequence, we examined the characteristics of circRNAs in FF exosomes from PCOS patients compared to those from women without PCOS, and we connected the differentially expressed circRNAs to the distinctive biological pathway.

In this study, we found circ_0008285, a non-coding RNA on chromosome 6 that was downregulated in PCOS patients by deep sequencing in the FF exosome of PCOS. On the other hand, there is no research on how circ_0008285 works in PCOS. Circ0008285, a circular RNA created from the CDYL gene's fourth exon, is primarily found in the cytoplasm and lacks an intronic sequence [12]. We sought to understand the function of circ_0008285 in PCOS and its underlying mechanism in this study. Furthermore, it was hypothesized that the molecular mechanisms underpinning the function of circ_0008285 in PCOS were mediated through the miR-4644/LDLR axis. As a result, this study might have revealed new details about aberrant lipid metabolism in PCOS.

## Materials and methods

### Individuals and samples

Samples were collected between January 2021 to May 2022 after patients provided written informed consent. The study was carried out following the Population and Family Planning Law of the People's Republic of China. It was authorized by the ethical committee of the Zhongshan Hospital, Fudan University (Shanghai, People's Republic of China).

Sixty-seven people (31 with PCOS and 36 controls) who underwent intracytoplasmic insemination (ICSI) with in vitro fertilization (IVF) at the Reproductive Center, Zhongshan Hospital, Fudan University, provided the FF for this study. These patients are distinctive and different from the patients in our previous PCOS research [13]. The control group got IVF for a male factor infertility reason. After removing patients with Cushing's disease, congenital adrenal hyperplasia, and androgen-secreting tumours, the diagnosis of PCOS was made using the Rotterdam updated criteria. Age ≥ 40 years, body mass index > 35 kg/m^2^, basal Follicle stimulating hormone (FSH) level > 12 mIU/mL, three or more prior unsuccessful IVF cycles, and systemic sickness or endocrine abnormalities were the exclusion criteria for the two groups.

### Treatment protocol

The antagonist stimulation method was administered to each patient. Briefly, on day 3 of the cycle, daily injections of recombinant FSH (r-FSH) (Gonal-F; Merck Serono) were used to initiate ovarian stimulation. An antagonist of gonadotropin-releasing hormone (GnRH) (Cetrotide, 0.25 mg; Merck Serono) began to use on day 6 of stimulation. An intramuscular injection of 5,000 IU of human chorionic gonadotropin (hCG) starts final oocyte maturation when at least three follicles have reached a diameter of 17 mm, or two have reached a diameter of 18 mm.

### Follicular fluid collection

The follicular fluid of the largest, first punctured follicle was collected during the oocyte retrieval procedure (transvaginal follicular aspiration). One follicle was collected per patient. All the follicles we selected are surrounded by the cumulus-oocyte complexes. Each ovarian follicle was aspirated independently. The diameter of the follicles was recorded at least three times by using vaginal B‐mode ultrasound before puncture and the average value was taken. The follicular fluid was collected from follicles with a diameter larger than 15 mm. The collected FFs were checked for erythrocytes. FFs with erythrocytes were excluded from the study.

### Exosome isolation

Ultracentrifugation was used to isolate the exosomes from the follicular fluid. Our previous study [13] characterized exosomes using transmission electron microscopy, nanoparticle tracking analysis, and western blot analysis. Follicular fluid was centrifuged at 300 g for 10 min to remove cells. The supernatant fluid was then centrifuged at 2000 g for 10 min at 4 °C to remove dead cells. The resultant supernatant fluid was transferred to an ultra-centrifuge tube and centrifuged at 100,000 g for 2 h. The pellet was suspended in PBS and filtered through a 0.22-μm filter, and then centrifuged at 100,000 g for 2 h. The pellet was resuspended in 200 μl PBS and stored at -80 °C. Runan Medical Technology (Suzhou) Ltd., Co. carried out the transmission electron microscopy and nanoparticle tracking detection procedures.

### RNA isolation, library construction, and sequencing

TRIzol reagent (Life Technologies, Carlsbad, CA, USA) was used to extract the exosome's total RNA. The NanoDrop ND-2000 (Thermo Scientific) was used to measure total RNA. KangChen Biotech (Shanghai, China) constructed the circRNA sequencing and RNA library construction. EdgeR package (http://www.rproject.org/) was used to find circRNAs that were differentially expressed between two groups. A circRNA candidate was deemed to have differential expression if at least a two-fold statistically significant variation in its levels between two experiment groups (*p* < 0.05). Based on the expression levels of all identified circRNAs and the significant difference between exo-PCOS and exo-control, the hierarchical clustering analysis was carried out by Cluster and TreeView software to generate the overview of differentially expressed circRNAs between exo-PCOS and exo-control.

### Grouping and treatment of ovarian granulosa cells

Human GCs (KGN cell) were purchased from Nuobai Biotechnology (Shanghai, China). All the cells were maintained at 37 °C under 5% CO2 in DMEM/F12 media supplemented with 10% fetal bovine serum (FBS) and 1% penicillin/streptomycin. Six groups of KGN cells were created: (1) the sh-NC group; (2) the sh-circ0008285 group; (3) the mimics-NC group; (4) the miR-4644 mimics group; (5) the sh-circ0008285 + inhibitors NC group; and (6) the sh-circ0008285 + miR-4644 inhibitors group. Among them, mimics NC, miR-4644 mimics, inhibitors NC, miR-4644 inhibitors, sh-NC, and sh-circ0008285 were all invented and synthesized by GenePharma Co., Ltd.(Shanghai, China). The cells were transfected when one well's confluence reached 60–70%. Using the Lipofectamine 2000 reagent (Invitrogen, Carlsbad, CA, USA), miRNA mimics, miRNA inhibitors, or plasmids were transfected into KGN cells. The cells were gathered 48 h post-transfection for further analysis.

### Luciferase reporter constructs and luciferase activity assay

The luciferase activity test was used to assess the direct contact between the partners of the circ_0008285-mediated ceRNA network. A luciferase reporter vector (GP-mirGLO Dual-Luciferase miRNA Target Expression Vector; Promega) was employed. Circ_0008285 and the 3' UTR of LDLR were cloned using RT-PCR. Circ_0008285-WT, circ_0008285-MUT and LDLR-3’UTR (WT and MUT) were constructed. Before transfection, KGN cells were seeded onto 24-well plates and given time to proliferate for 24 h without using antibiotics. Using Lipofectamine 2000, the created reporter vectors (300 ng) were transfected into cells with the miRNA (miR-4644) mimics or negative control mimics. The Dual-Luciferase Reporter Assay System was used to measure the luciferase activity in lysed cells 24 h after transfection (Promega). Activities of Renilla luciferase were converted to those of Firefly luciferase.

### Western blot analysis

Western blot analysis was used to measure CD9, TSG101, Calnexin, and LDLR expression, and the samples were normalized to tubulin. RIPA buffer was used to prepare the total protein. PVDF membranes (EMD Millipore, Billerica, MA, USA) were used to transfer the protein lysate that had been separated by 12% SDS-PAGE. The membranes were blocked for one hour, incubated overnight at 4 °C with primary antibodies against CD9 (13403S, 1:1000, CTS), TSG101 (ab125011, 1:1000, Abcam), Calnexin (ab125011, 1:1000, Abcam), LDLR (ab52818, 1:1000, Abcam), and beta-Tubulin (T0023, 1:1000, Affinity Biosciences), and then incubated with the corresponding secondary antibodies at 1:2000 dilution for 2 h at room temperature. Horseradish peroxidase (HRP)-conjugated goat anti-rabbit IgG (H + L) (#A0208, 1:2000 dilution, Beyotime) was the secondary antibody against CD9, TSG101, Calnexin and LDLR. HRP-conjugated goat anti-mouse IgG (H + L) (#A0216, 1:2000 dilution, Beyotime) was the secondary antibody against beta-Tubulin.

### Gene expression analysis

The expression of Pparg and Pgc1 was examined to evaluate lipid metabolism. The mRNA expression of the limiting enzyme, 3-hydroxy-3-methyl glutaryl-CoA reductase (Hmgcr), was used to evaluate de novo cholesterol synthesis. Real-time PCR was used to measure all mRNA levels, and a list of the primers used in real-time PCR is provided in Supplemental Table 4.

### Lipid profiles analysis

Total cholesterol (TC), High-density lipoprotein (HDL), Low-density lipoprotein (LDL), Free fatty acid (FFA), and total triglycerides (TG) lipid profiles of the culture medium of KGN cells were determined using immunoassays. TC, HDL, LDL and TG were detected in Roche Cobas C702 machine by enzyme-induced colorimetry (TC:#05168538190, Roche; HDL:#05168805190, Roche; LDL:#07005768190 Roche; TG:#05171407190, Roche). FFA was detected in Hitachi 7600 automatic biochemical analyzer. FFA: #157,819,910,930, Desai Diagnosis System (Shanghai).

### Statistical analysis

SPSS (Chicago, Illinois, USA) was utilized for statistical analysis. Groups were compared using the t-test for normally distributed variables and the Mann–Whitney U test for not-normally distributed variables. The difference between the various groups was evaluated using the two-tailed unpaired t-test. Means and SEM were used to display the variables. Statistical significance was defined as *p* < 0.05 for each and every comparison.

## Results

### Isolation and identification of exosomes

Exosomes were extracted from the follicular fluid of PCOS and control patients using ultracentrifugation. Transmission electron microscopy (TEM) demonstrated that FF exosomes had rounded morphologies and sizes ranging from 40 to 100 nm (Supplemental Fig. 1A). Additionally, western blot analysis verified the exosome markers CD9 and TSG101 were present (Supplemental Fig. 1B). The size distribution determined by NTA also revealed a typical exosome profile (Supplemental Fig. 1C). The findings from TEM, NTA, and Western blot were in line with exosome features.

**Fig. 1:**
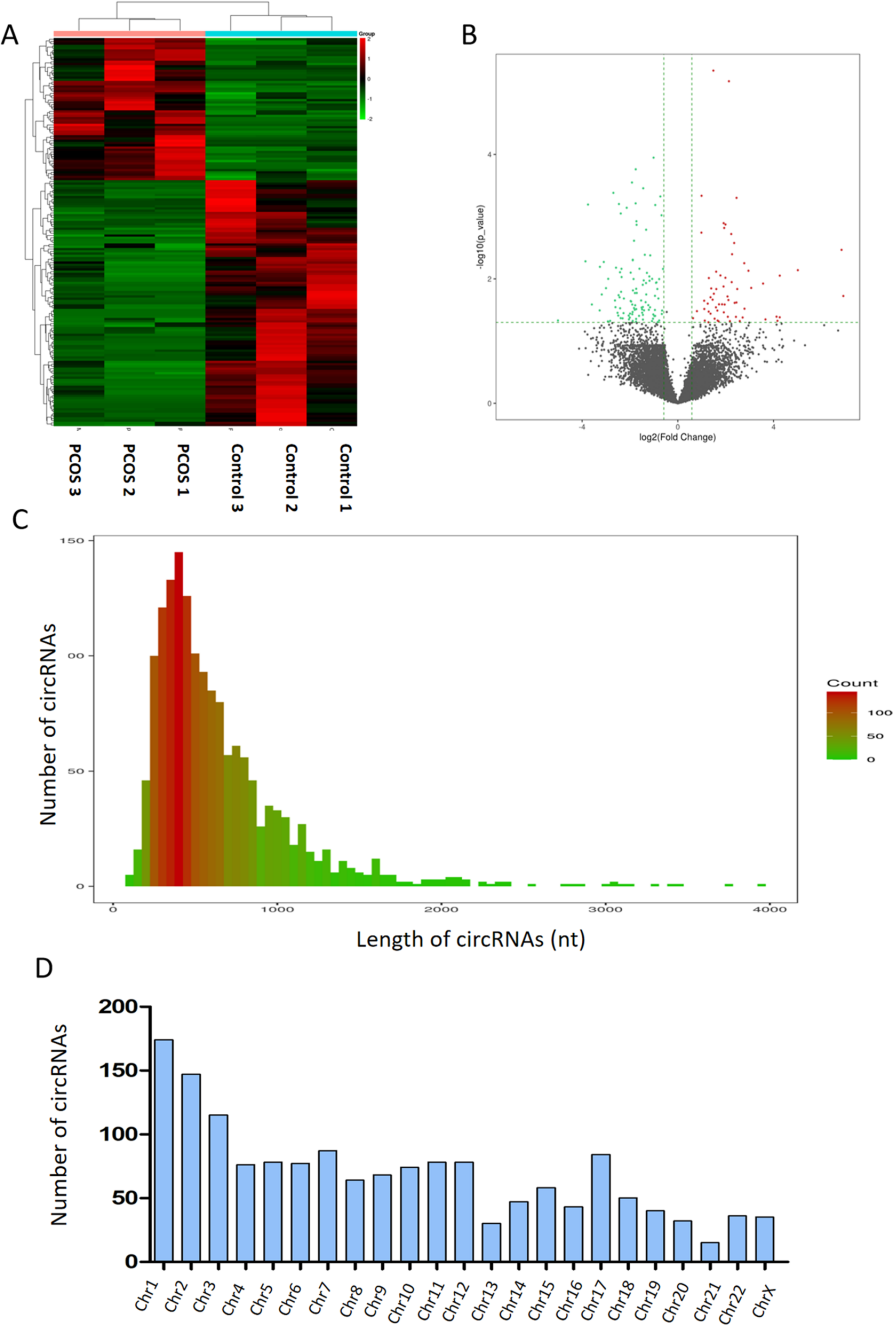
Differential expression of circular RNAs between polycystic ovary syndrome and control samples. **A** Supervised hierarchical clustering of the PCOS and control groups. Red = up- regulated; green = down-regulated. **B** Volcano plots visualizing the differentially expressed circRNAs. The red and blue plots represent the statistically significantly up- and down-expressed circRNAs respectively. A schematic overview of the circRNA sequencing results showing the distribution of circRNAs based on length (**C**) and chromosomal location (**D**)

### Exosomal circRNA expression differences between PCOS patients and controls

RNA sequencing was used to analyze the circRNA expression profiles of exosomes isolated from FF in patients with PCOS (*N* = 3) and controls (*N* = 3). In Supplemental Table 1, the clinical data of PCOS patients and non-PCOS donors were displayed. Comparison of the exosomes extracted from FF with two databases revealed a total of 1585 circRNAs (circBASE and CIRCexplorer). Unsupervised hierarchical clustering analysis was used to assess the difference in circRNA expression profiles between the two groups. The results showed that 3 PCOS patients' circRNA expression patterns differed statistically substantially from the 3 patients in the control group (Fig. 1A). The change in circRNA expression between the two groups was also displayed by the volcano plots (Fig. 1B). Supplemental Table 2 displays the names, fold change, and P value of the top ten differentially expressed circRNAs. The total number of circRNAs had a mean length of 525 and a mean length of 526 nucleotides (nt), respectively. In addition, the mean lengths of the other circRNA isoforms were shown in Fig. 1C. CircRNAs were frequently found to be widely distributed across all of the chromosomes. Over 100 circRNAs were produced by chromosome 1, chromosome 2, and chromosome 3, while the majority of the other chromosomes produced between 15 and 100 circRNAs. Figure 1D depicts the various circRNA types' distribution throughout the human genome.

### qRT-PCR validation of potential circRNAs

**Fig. 2:**
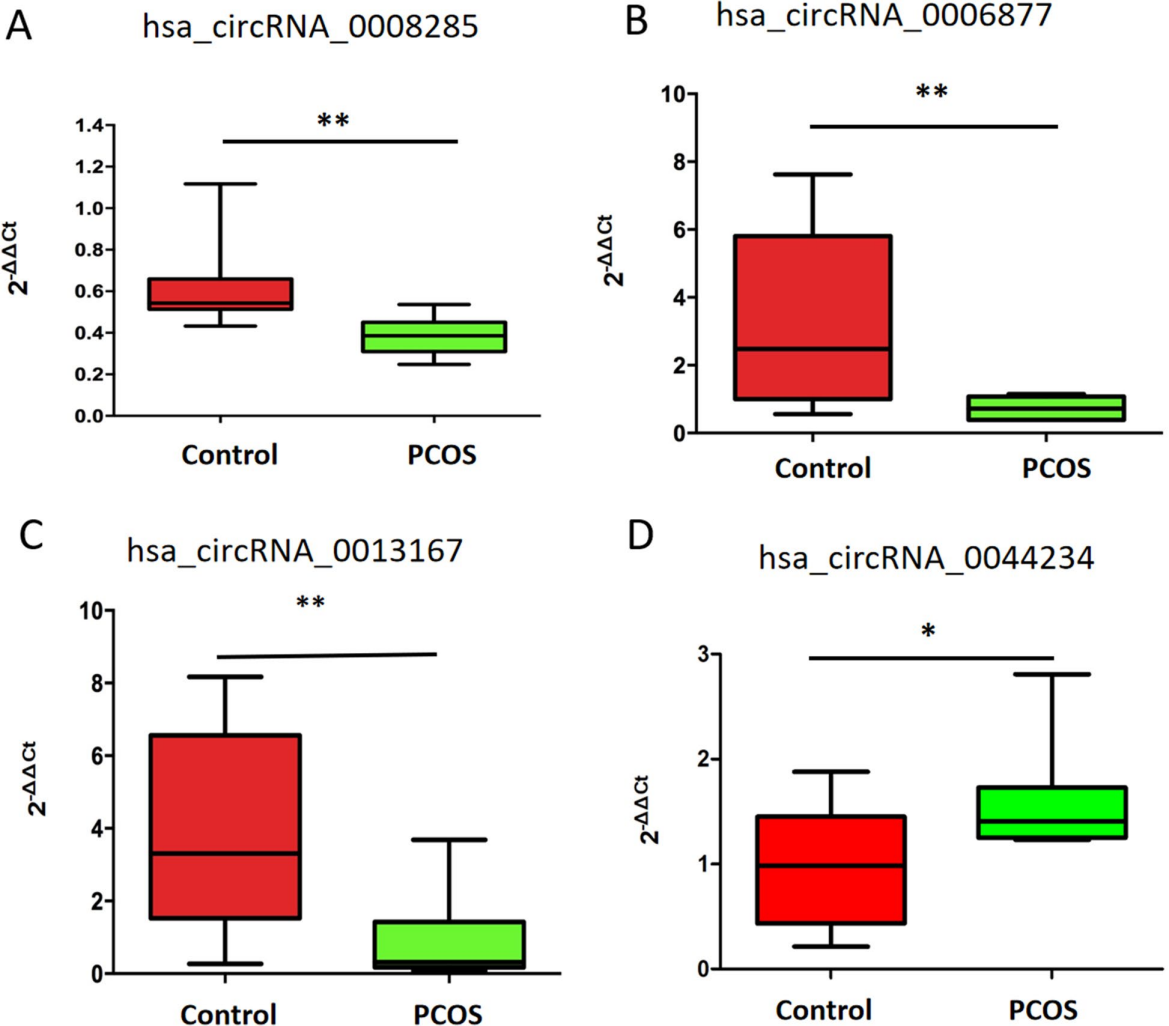
Quantitative real-time polymerase chain reaction validation of selected circular RNAs. Validation by qRT-PCR of four aberrantly expressed circRNAs in the PCOS group compared with the control group. *P < 0.05, **P < 0.01

Using exosomes of FF obtained from an additional 28 PCOS patients and 33 non-PCOS patients, the expression of hsa_circ_0044234, hsa_circ_0008285, hsa_circ_0013167, and hsa_circ_0006877 were evaluated by qRT-PCR to confirm the findings. Supplemental Table 4 lists the primers utilized for the qRT-PCR study of these four circRNAs. According to the RNAseq results, the relative expression levels of the four circRNAs were different in the PCOS group compared to the control group. Circ_0044234 was overexpressed in PCOS patients, while circ_0006877, circ_0013167 and circ0008285 were decreased in PCOS (Fig. 2).

### Bioinformatics analysis of circ0008285

**Fig. 3:**
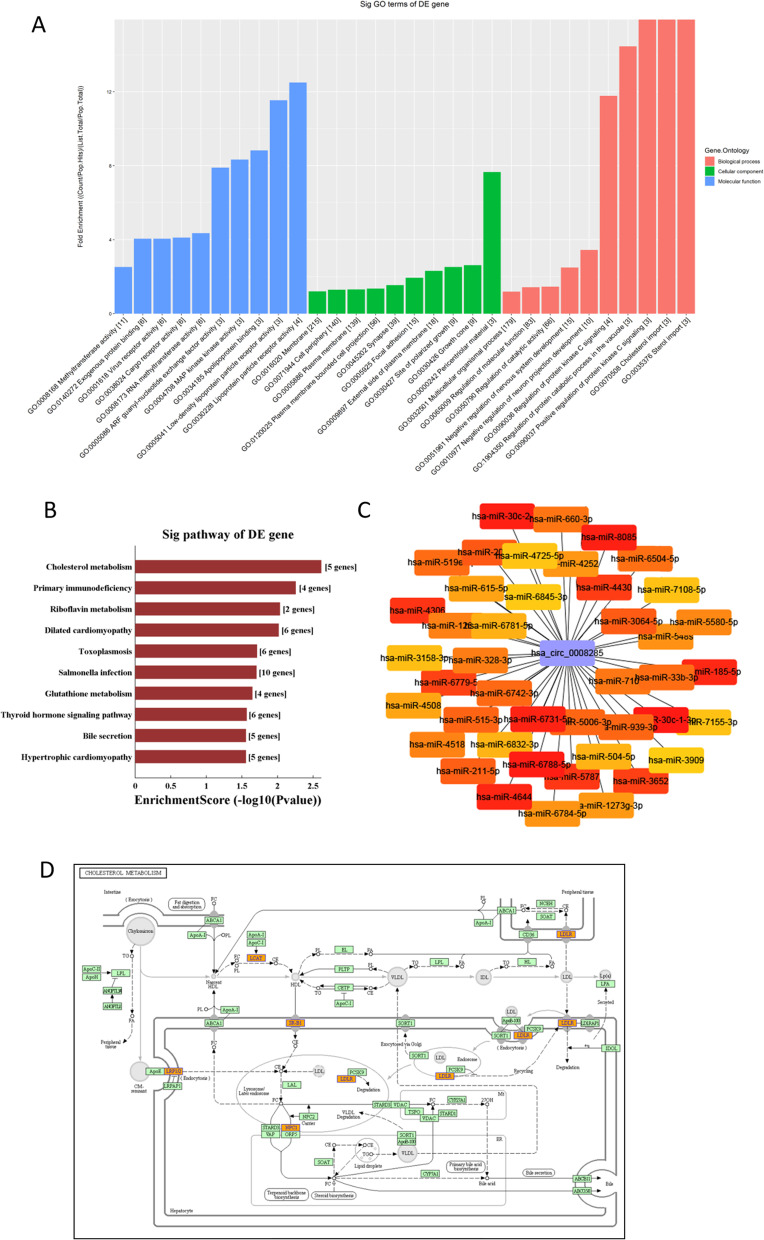
The predicted function, pathways and regulatory network of circ_0008285. **A** Top 10 GO terms of the gene enrichment analysis of circ_0008285 for cellular components, biological processes, and molecular functions, respectively. **B** The top 10 enriched KEGG pathways of circ_0008285 parental genes. **C** The circRNA-miRNA gene network of circ_0008285. **D** The diagrammatic sketch of cholesterol metabolism pathway circ_0008285 involved

The GO enrichment analysis of differently functioning biological processes, cellular components, and molecular activities was carried out to assess the functional relevance of circ_0008285, and the enrichment score was represented as the log (P value). The most statistically substantially enriched phrase for the cellular component was pericentriolar material (GO: 0000242). For the biological process, the most statistically significantly enriched term was sterol import (GO: 0035376). As for the molecular function, the term with the most predominantly enriched genes was lipoprotein particle receptor activity (GO: 0030228). Meanwhile, cholesterol metabolism was the most statistically enriched pathway in the KEGG pathway analysis. These biological processes and pathways are tightly connected to PCOS development (Fig. 3A&B&D). Recent researches have shown that circRNAs can act as miRNA sponges to control gene expression by persistent complementary binding and miRNA absorption [14, 15]. Using miRNA response elements (MREs), we discovered the potential miRNAs that could bind to circRNAs. The circRNA-miRNA gene network was created and visualized using the Cytoscape software to illuminate the relationships (Fig. 3C).

### Characterization of circ_0008285

**Fig. 4:**
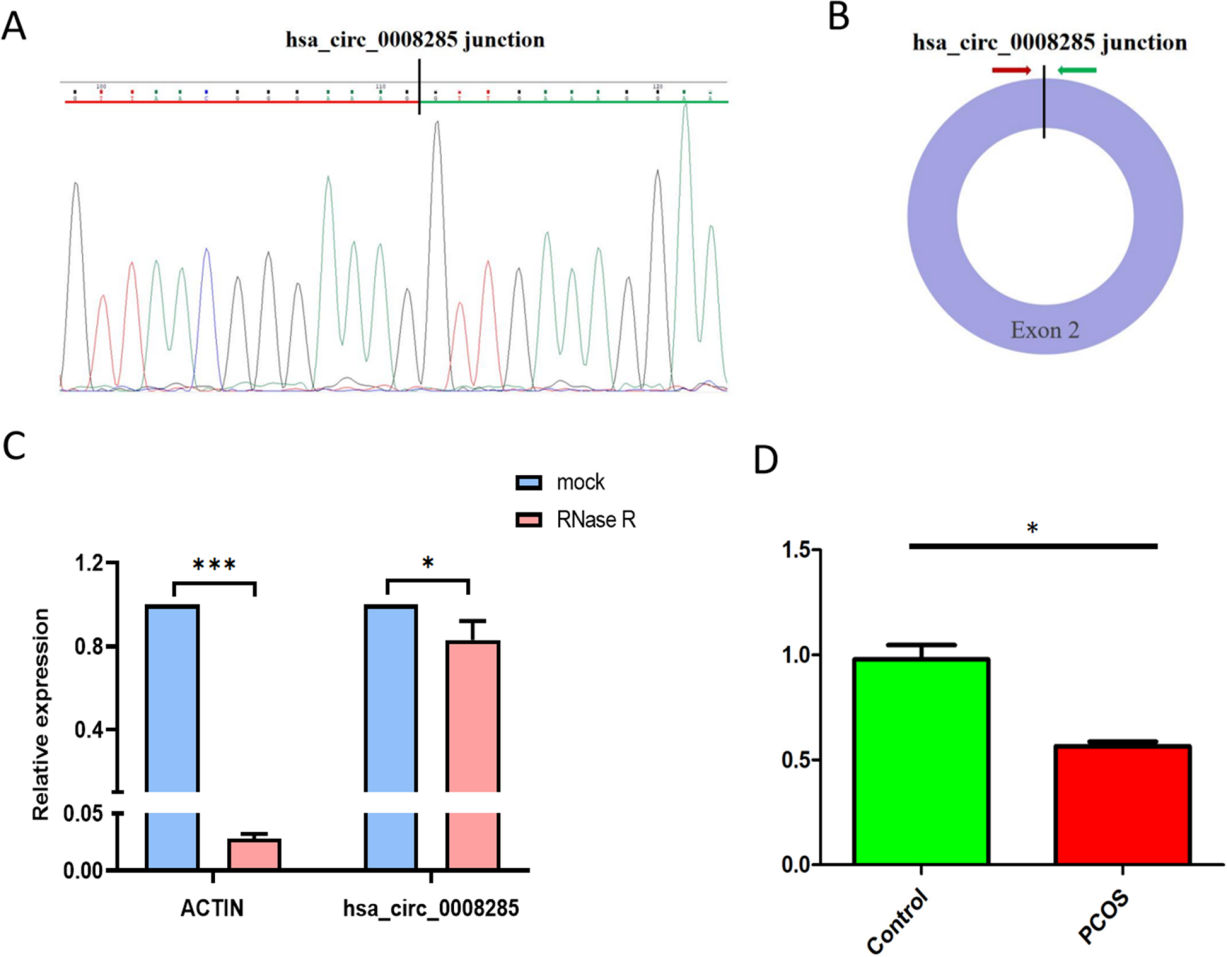
The characteristics of circ_0008285. **A** The annotated region in CDYL gene for the formation of circ_0008285 was shown. **B** The exact sequence of the back splicing site in circ_0008285 was confrmed by sequencing. **C** The RNase R tolerance test proved the circular format of circ_0008285. **D** Quantitative PCR analysis of the expression levels of circ_0008285 in KGN cells after normal-FF-exos or PCOS-FF-exos treatment for 48 h. All data are presented as the mean ± SD. Student’s t-test was used for statistical analysis. *P < 0.05, ***P < 0.005

Circ_0008285 originates from the Chromodomain Y Like (CDYL) gene located on chr6:4,776,680–4,955,778, which is generated by back splicing of exon 2 and has a length of 678 bp. Sanger sequencing of the PCR results was used to confirm the location of the circ_0008285 back splicing junction point (Fig. 4A&B). Circ_0008285 was resistant to RNase R in the RNase R digestion experiment. In contrast, the linear isoform was reduced due to RNase R treatment (Fig. 4C). In KGN cells treated with PCOS-FF exosomes as opposed to normal-FF exosomes, circ_0008285 levels were also significantly increased (Fig. 4D).

### Circ0008285 is a sponge of miR-4644

**Fig. 5:**
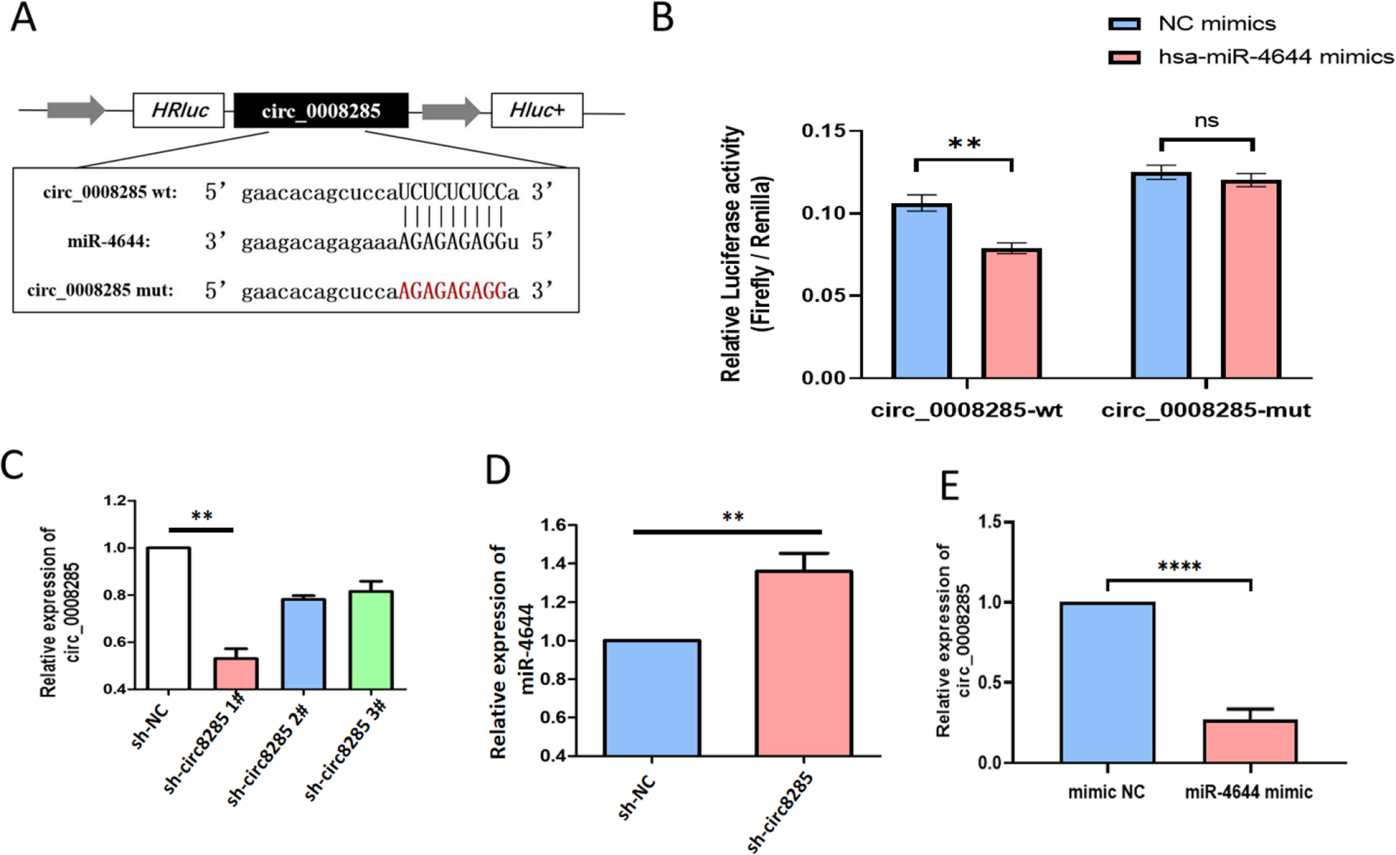
miR-4644 was negatively regulated by circ_0008285. **A** Direct binding sites of miR-4644 in WT 3ʹ-UTR of circ_0008285 and MUT 3ʹ-UTR of circ_0008285 were designed. **B** WT circ_0008285 or MUT circ_0008285 recombinant plasmids and miR-4644 mimic or mimic nc were co-transfected and luciferase reporter assay was performed. **C** The expression level of circ_0008285 in KGN cells when transfected with three HUPCOS shRNAs. **D** miR-4644 levels were detected using RT-PCR in KGN cell knockdown of circ_0008285. **E** circ_0008285 levels were detected using RT-PCR in KGN cell with miR-4644 mimic. *P < 0.05, **P < 0.01, ****P < 0.001

According to bioinformatics research, the WT 3'-UTR of circ_0008285 was able to partially bind to miR-4644 rather than the MUT 3'-UTR of circ_0008285 (Fig. 5A). In KGN cells expressing the WT 3'-UTR of circ_0008285, miR-4644 mimic transfection resulted in a reduction in luciferase activity (*P* < 0.01) as compared to mimic-nc transfection. However, the cells expressing the MUT 3'-UTR of circ_0008285 were unaffected by luciferase activity (Fig. 5B). Therefore, we depleted circ_0008285 expression in KGN cells using short hairpin RNA to address the potential role of circ_0008285. The efficiency of the shRNA transfection was confirmed by qRT-PCR. Of the three shRNAs tested, circ_0008285 shRNA 1# caused a considerable drop in circ0008285 level when compared to control (Fig. 5C). Knockdown of miR-4644 raised the levels of circ_0008285 (Fig. 5D). Compared to negative control mimics, miR-4644 mimics significantly reduced mRNA expression of circ_0008285 (Fig. 5E). These results indicate that miR-4644 directly binds to circ_0008285.

### LDLR is a target of miR-4644

**Fig. 6:**
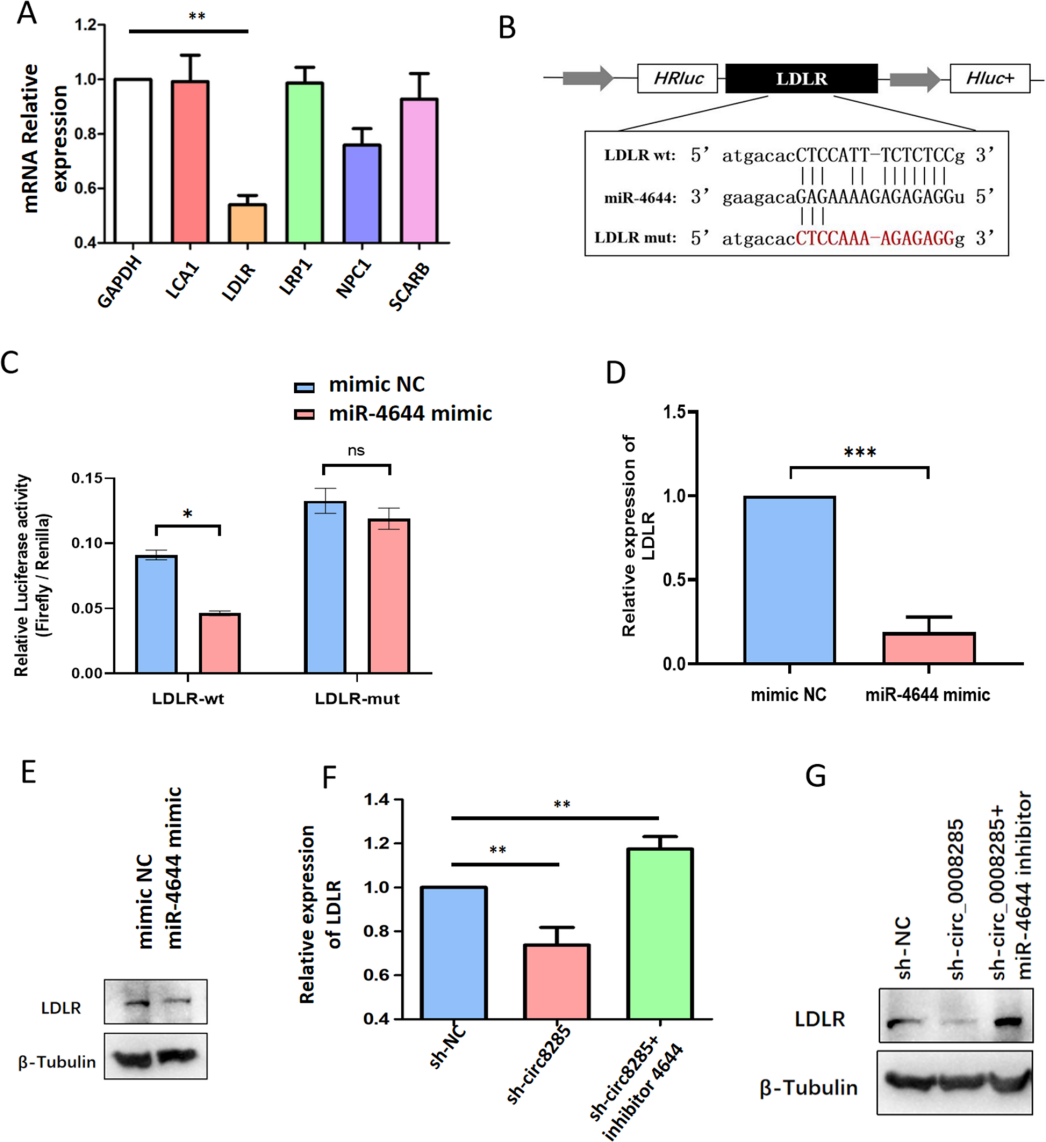
miR-4644 directly targeted downstream LDLR. **A** Downstream mRNAs expression levels after silencing circ_0008285. **B** Direct binding sites between miR-4644 and WT 3ʹ-UTR of LDLR are shown. MUT 3ʹ-UTR of LDLR was also designed. **C** Luciferase activity was tested in KGN cells transfected by WT LDLR or MUT LDLR recombinant plasmids and miR-4644 mimic or mimic nc. **D** LDLR mRNA expression was determined in miR-4644 mimic cells. **E** LDLR was measured using western blotting in miR-4644 mimic cells. LDLR mRNA expression (**F**) and protein level (**G**) were determined in KGN cells. *P < 0.05, **P < 0.01, ***P < 0.005

In addition, LDLR was a potential target of miR-4644 (Fig. 6A). We also created luciferase reporter vectors by cloning WT or mutant (lack of miR-4644 binding site) 3'-UTR of LDLR downstream of the Renilla luciferase gene and transfected them with miR-4644 mimics into the KGN cells to ascertain whether LDLR is a direct target of miR-4644. The relative luciferase activity in KGN cells co-transfected with the LDLR WT constructs was considerably reduced by miR-4644 mimics. The miR-4644 binding site in 3’-UTR is necessary for such regulation, as shown by the LDLR mutant constructs (Fig. 6B). Thus, miR-4644 suppressed the expression of LDLR by directly binding to its 3’UTR (Fig. 6C). The expression of LDLR was dramatically suppressed by miR-4644 overexpression (Fig. 6D&E), whereas LDLR was promoted by miR-4644 inhibition (Fig. 6F&G).

### miR-4644 targets LDLR to influence cholesterol metabolism

**Fig. 7:**
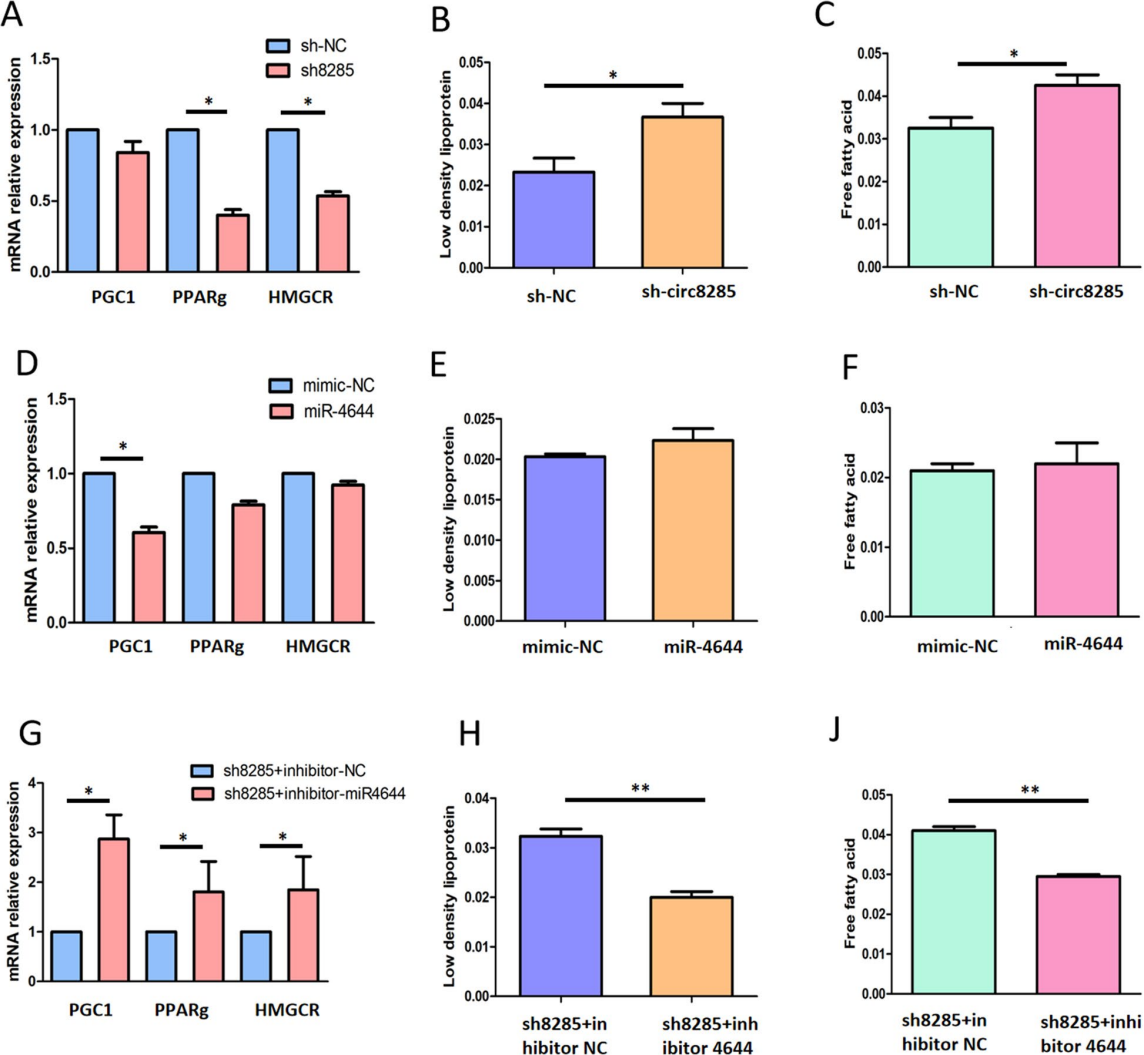
miR-4644 targets LDLR to influence cholesterol metabolism. **A** The mRNA expression of Pparg, Pgc1 and Hmgcr in sh-NC and sh-circ_0008285 group. **B** & **C** The effects of circ_0008285 on LDL and free fatty acid production in sh-NC and sh-circ_0008285 group. **D**The mRNA expression of Pparg, Pgc1 and Hmgcr in mimic-NC and mimic-miR-4644 group. **E** & **F** The effects of circ_0008285 on LDLand free fatty acid production in mimic-NC and mimic-miR-4644 group. **G** The mRNA expression of Pparg, Pgc1 and Hmgcr in sh-circ_0008285 + inhibitor-NC and sh-circ_0008285 + inhibitor-miR-4644 group. **H** & **J** The effects of circ_0008285 on LDL and free fatty acid production in sh-circ_0008285 + inhibitor-NC and sh-circ_0008285 + inhibitor-miR-4644. *P < 0.05, **P < 0.01

The subsequent effects of these modeled exosomes on the recipient KGN cells were then investigated. The most frequent clinical manifestation of polycystic ovary syndrome (PCOS) is lipid metabolic and ovarian disorders. Affecting the local microenvironment of follicular fluid, abnormalities in lipid metabolism can either directly or indirectly affect the growth and function of follicles. We further analyzed the expression of Pparg, Pgc1 and Hmgcr and the LDL and free fatty acid metabolism. As a master regulator of adipogenesis and a ligand-activated transcription factor, PPARg regulates genes involved in inflammation, bone biology, and lipid homeostasis [16]. In granulosa cells, PGC1, a transcriptional coactivator with PPARg, controls the synthesis of steroid hormones [17]. Hmgcr is a crucial enzyme in the de novo synthesis of cholesterol. We found a decrease in PPARg and HMGCR mRNA (Fig. 7A) in the sh-circ0008285 cells compared to the control. Low-density lipoprotein and free fatty acid were increased in the sh-circ0008285 cells (Fig. 7B&C). Moreover, PGC1a mRNA (Fig. 7D) was decreased in the miR-4644 mimic cell. Low density lipoprotein and free fatty acid were not changed in miR-4644 cells (Fig. 7E&F). In contrast to the sh-circ0008285 + inhibitors NC group, Pparg, Pgc1 and Hmgcr expression were increased in KGN cells of the sh-circ0008285 + miR-4644 inhibitors group (Fig. 7G). LDL and FFA of the sh-circ0008285 + miR-4644 inhibitors group were dramatically increased (Fig. 7H&I). The results indicated that fatty acid metabolism was also impaired by circ_0008285.

## Discussion

Process of foliculogenesis requires interactions between endocrine and intra-ovarian paracrine systems to provide the ideal microenvironment for oocyte development [18]. The follicular fluid (FF), which builds up inside the antral follicle, is a crucial milieu for the oocyte's development, and its biochemical makeup reveals the follicle's physiological state [19]. The majority of cells secrete exosomes which is a tiny membrane bubble. It has a lipid bilayer membrane structure with a diameter of roughly 40–100 nm, which contains cell‐specific proteins, lipids, and nucleic acids including DNA, mRNAs, microRNAs (miRNAs), circular RNAs and long noncoding RNAs. Exosomes have enormous potential as therapeutic agents and diagnostic biomarkers for a wide range of pathophysiological ailments, including neurological illnesses, persistent malignancies, infectious diseases, female reproductive disorders and cardiovascular diseases [20].

To date, there have only been a few investigations on follicular fluid exosomes in PCOS patients. According to Lilian Bai et al. [4], fatty acid metabolism, peroxisome proliferator-activated receptor (PPAR) signaling pathways, and lipid metabolism were significantly enriched in the various expression genes of follicular fluid exosomes between PCOS and control. The follicular fluid exosomal miRNAs hsa-miR-196a-3p, hsa-miR-143-5p, hsa-miR-106a-3p, hsa-miR-34a-5p, and hsa-miR-20a-5p were possible biomarkers for the diagnosis of PCOS, as revealed by Ye Tian-Min et al. [21]. Depleting circLDLR in exosomes raised the expression of miR-1294 and decreased the expression of CYP19A1 in recipient cells, according to Xin Huang [22], who also offered fresh details on the aberrant follicle development in PCOS. Our study also verified circLDLR in PCOS, which was consistent with Xin Huang’s study. Besides that, we verified another three different circRNAs in follicular fluid exosomal in PCOS. However, the role of FF exosomes in PCOS pathological follicle development has yet to be clarified.

Circular RNAs (circRNAs), non-coding RNAs produced by splicing, have been linked to several biological processes, including the sequestration of proteins, enhanced parental gene expression, and translation into polypeptides [23]. Che et al. [24] determined 311 increased and 721 decreased circRNAs in cumulus cells from PCOS patients compared to control subjects who underwent IVF. Healthy ovarian cortex from young (25–28) and aged (44–46) individuals were compared for circRNA expression profiles by Cai et al. [25]. 194 upregulated and 207 downregulated circRNAs were enriched in oxidation–reduction, steroid hormone production, and insulin secretion pathways throughout aging. According to Hongcai Cai [26], circDDX10 levels in GCs steadily declined with aging and were positively linked with AMH and AFC. CircDDX10 was found to be a unique biomarker for predicting ART outcomes and was connected to the quantity of oocytes obtained and good-quality embryo rates. Ai-Xue Chen et al. [11] showed that circ_0043533 decreased the silence on cell proliferation and apoptosis in ovary-related cells by acting as a sponge to absorb miR-1179. In their investigation of the role of circ_RANBP9 in PCOS, Xiaohui Lu et al. [27] found that its silencing decreased GC growth and promoted apoptosis via the miR-136-5p/XIAP pathway. However, there are no reports on the role of circ_CDYL (hsa_circ_0008285) in PCOS. A previous study [28] revealed that circ_0008285 expression is elevated in HCC tissues and related to the proliferation, migration and invasion of HCC cells. Additionally, Rui Zhou et al. [12] showed that circ_CDYL was downregulated in Wilms' tumor tissue. CircCDYL, functioning as a miRNA sponge, facilitated the circCDYL/miR-145-5p/TJP1 axis. In the current study, we found that circ_0008285 levels were decreased in the FF exosome of patients with PCOS. According to the GO and pathway analysis of circ_0008285, the parental genes are enriched in lipoprotein particle receptor activity and the cholesterol metabolism pathway was the most significantly altered pathway. The results are consistent with the fact that PCOS is accompanied by the repression of gene signatures associated with cholesterol and lipids [29].

Bioinformatics analysis in this study indicated miR-4644 as the target of circ_0008285, and luciferase reporter experiments further confirmed the direct interaction between circ_0008285 and miR-4644 in KGN cells. Although the role of miR-4644 in follicle development was unclear, previous studies had reported that miR-4644 could be the target of lncRNAs (i.e.lncPVT1 [30], lncELFN1-AS1 [31]) and regulate cell proliferation and migration. Here, we present unique data demonstrating that miR-4644 was directly downregulated in PCOS lipid metabolism by circ_0008285.

It has been established that the low-density lipoprotein receptor (LDLR) is crucial for lipoprotein metabolism. Compared to the control group, LDLR^−/−^mice had significantly fewer follicles, greater follicular atresia, lower estrogen levels, and shorter estrus duration [32]. In our study, we demonstrated that LDLR was a target of miR-4644. The high level of LDLR protein level cause the reduction in free fatty acid and low-density lipoprotein and increases the mRNA expression of Pparg, Pgc1 and Hmgcr. We also found a significant positive correlation between the expression of circ_0008285 and LDLR. Furthermore, the critical withdrawal experiments and luciferase assay in KGN cells proved that LDLR was involved in the circ_0008285/miR-4644 pathway as a crucial enzyme inhibiting lipid metabolism.

It is common knowledge that changes in metabolic status, like obesity and dyslipidemia, may have a negative effect on female fertility [33]. Programming lipid pathways can occur in a variety of tissues, including reproductive ones [34]. The nuclear receptors for the peroxisome proliferator-activated receptors (PPARs) control steroidogenesis, intracellular lipid metabolism, and oxidative stress. Female subfertility has been linked to disruptions of this system. PGC1 modulates the synthesis of steroid hormones in granulosa cells. PPARg, the master regulator of lipid metabolism, also regulates steroidogenic enzymes and is involved in female reproductive functions [35]. As a substrate for steroid production and energy source, fatty acid and cholesterol metabolism play an important role in steroidogenesis. Here, we report that lipid pathways and metabolism were affected by alterations on the expression of circ_0008285 and LDLR.

Since the number of circRNAs that have been identified is rapidly increasing, additional research will be required to investigate their molecular and biological functions. However, in order to obtain a complete picture of the circ0008285/miR-4644/LDLR axis in PCOS, additional studies will be required, such as an in-depth investigation of the mechanism with a larger sample and a promising clinical application in PCOS therapeutic treatment.

## Conclusion

In summary, the down-regulated circ_0008285 was suggested to be involved in abnormal ovarian lipid metabolism, and the altered exosomal circRNAs profiles that were found in PCOS follicular fluid exosomes were discussed. We also showed that the circ_0008285 in exosomes was a crucial mediator that was sponging miR-4644 to repress LDLR controlled lipid secretion. The molecular processes involved in PCOS can now be better understood thanks to our findings, which could lead to new targets and approaches for PCOS treatments.

## Supplementary Information

**Additional file 1.** 

**Additional file 2.** 

**Additional file 3.** 

**Additional file 4.** 

**Additional file 5.** 

## Data Availability

The data supporting the study are available to the corresponding author upon reasonable request.
