# Supplementary figures and images for: Exosomal circ_0008285 in follicle fluid regulates the lipid metabolism through the miR-4644/ LDLR axis in polycystic ovary syndrome
Source: J Ovarian Res. 2023 Jun 15;16:113. doi: 10.1186/s13048-023-01199-x (PMC10268507; doi:10.1186/s13048-023-01199-x)

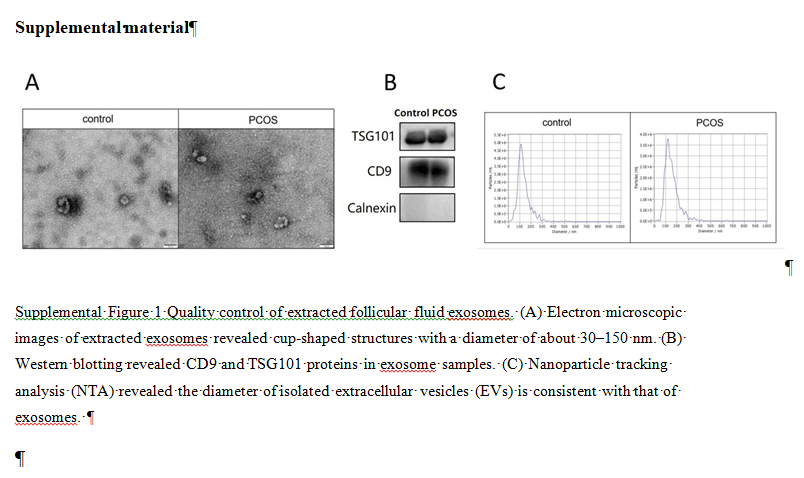

Supplement: Supplementary file 1 — Additional file 1. [file 13048_2023_1199_MOESM1_ESM.png]

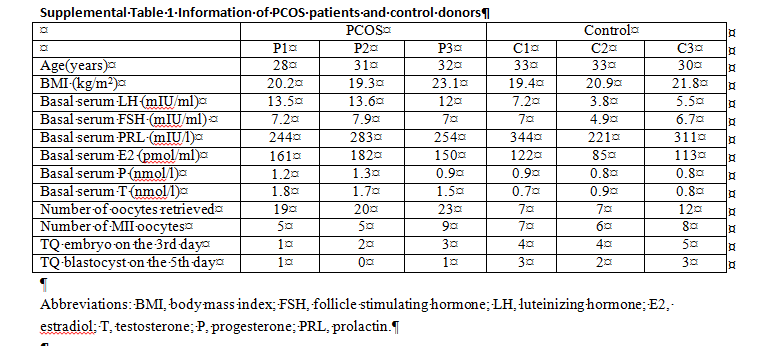

Supplement: Supplementary file 2 — Additional file 2. [file 13048_2023_1199_MOESM2_ESM.png]

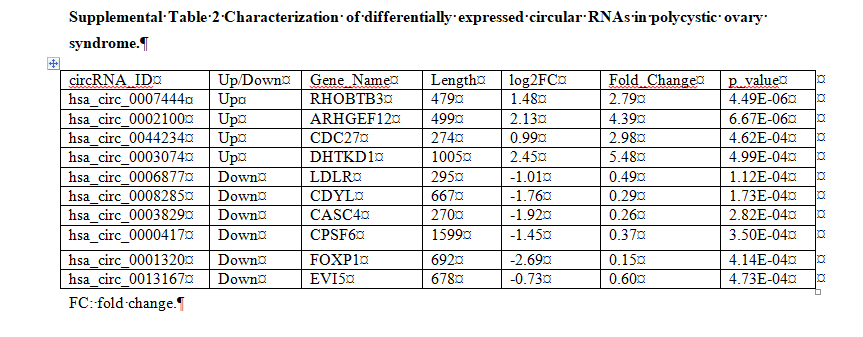

Supplement: Supplementary file 3 — Additional file 3. [file 13048_2023_1199_MOESM3_ESM.png]

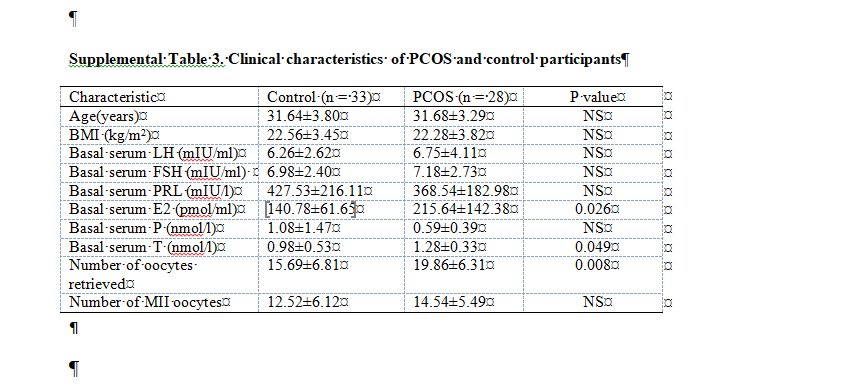

Supplement: Supplementary file 4 — Additional file 4. [file 13048_2023_1199_MOESM4_ESM.png]

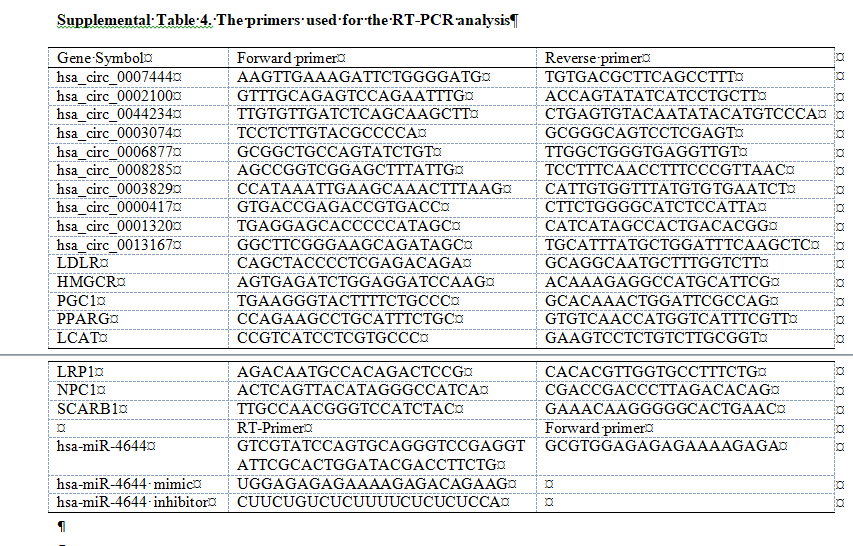

Supplement: Supplementary file 5 — Additional file 5. [file 13048_2023_1199_MOESM5_ESM.png]
